# Free-Living Gait Cadence Measured by Wearable Accelerometers for Assessing Fall Risk

**DOI:** 10.1093/geroni/igab046.1302

**Published:** 2021-12-17

**Authors:** Jacek Urbanek, David Roth, Marta Karas, Amal Wanigatunga, Stephen Juraschek, Lawrence Appel, Jennifer Schrack

**Affiliations:** 1 Johns Hopkins School of Medicine, Baltimore, Maryland, United States; 2 Johns Hopkins University, Baltimore, Maryland, United States; 3 Johns Hopkins Bloomberg School of Public Health, Baltimore, Maryland, United States; 4 Harvard Medical School, Boston, Massachusetts, United States

## Abstract

Accelerometers are widespread in research applications, yet whether they are superior to structured clinic-based assessments is unknown. Using negative binomial regression, we compared traditional in-clinic measures of mobility (6-minute gait cadence, speed, and distance, and 4-meter gait speed) with free-living gait cadence from wrist accelerometers (Actigraph GT9X) in predicting fall rates in 432 older adults (age 77.29±5.46 yrs, 59.1% men, 80.2% White) participating in the Study to Understand Fall Reduction and Vitamin D in You (STURDY). Accelerometry-based gait cadence was estimated with the Adaptive Empirical Pattern Transformation algorithm. Across all participants, every 10 steps/min higher cadence was associated with a 13.2% lower fall rate (p=0.036). Mobility measures were not related to falls (p>0.05). Among higher-functioning participants (cadence ≥100 steps/min), every 10 steps/min higher free-living cadence (p=0.01) was associated with a 27.7% lower fall rate. Data collected from accelerometers may provide a more sensitive indicator of fall risk than in-clinic tests.

